# Raising the Bar for Levees

**DOI:** 10.1289/ehp.114-a44

**Published:** 2006-01

**Authors:** Tim Lougheed

Human beings have likely been battling rising waters since the dawn of organized agriculture. Farmers around the world have traditionally been drawn to the rich soils of floodplains, which are generally well worth the trouble occasionally caused by surrounding waterways. Densely populated urban areas subsequently grew up around many of these same places, attracted by additional assets such as access to fishing and easy navigation. These settlements often require substantial and ongoing engineering efforts to secure the physical safety of the community. While the fundamental principles and challenges of holding back water have not changed, the tools we can bring to the task continue to become more sophisticated.

As events in the Gulf Coast recently demonstrated, efforts to hold back the sea are sometimes doomed to failure. Engineers are debating how and even whether the levee system around the New Orleans area should be rebuilt. But the options today are much greater than when the Mississippi River levees were first built.

Levees built today may look the same as they always have but can incorporate design, construction, and maintenance innovations that are finding their way into civil engineering. Some of these features smack of high technology, such as elaborate sensors to detect stresses and strains within the structure, so as to provide a warning of critical pressures that could signal serious damage or collapse. Similarly, impermeable lining materials known as geomembranes can be laid down underneath the structure before it is built, so that the seepage of water through the ground cannot erode foundations.

Above all, engineers continue to improve their understanding of water flows, taking advantage of ever more detailed computer modeling techniques to describe the implications of barrier design to experts in the field, political or legal authorities who may be responsible for those barriers, and members of the public.

## Lessons from the Dutch

Perhaps no country has a more vested interest in levee safety than the Netherlands, which has occasionally paid a high price for sustaining major population centers well below the level of the stormy North Sea. In the winter of 1953, the sea breached a system of dikes that had been in place since the Middle Ages, causing floods that killed nearly 2,000 people. This catastrophe galvanized the nation’s political and social commitment to mounting and maintaining a sophisticated system of barriers that has set the standard for the rest of the world.

From the 1950s to the 1980s, major dams were constructed to hem in hundreds of miles of the country’s vulnerable coastline, knit together with earthen embankments and massive sluice gates over the delta stretching across the mouths of the Rhine, Maas, Waal, and Schelde Rivers, which all drain into the North Sea. The scale of this project—dubbed the Delta Works—is highlighted by the Oosterschelde storm surge barrier, which was completed in 1986. Designed to protect the ecological integrity of the surrounding estuary, the structure features 62 openings for tides to flow back and forth.

Engineers had never before attempted to erect sea defenses on this scale, and the Dutch became pioneers in the field. The five-mile-wide opening at the Oosterschelde, for example, called for 65 separate concrete piers more than 100 feet in height, which were built in place to an accuracy on the order of a few inches. Such precision was ensured by setting them on gigantic steel mesh “mattresses” filled with sand and gravel, which would prevent erosion that could shift the piers out of position.

In 1997 an even more ambitious undertaking was completed in the country’s southwest, where the Maeslant flood barrier includes two hollow arched doors, each about 1,000 feet long and 70 feet high, which float in side channels when not in use. They are rotated into their protective posture by steel ball joints 35 feet in diameter. Once the gates meet in the middle, they fill with water and sink onto a concrete pad, effectively blocking any storm surge.

These engineering marvels are based on earlier measurements of river floods and storm surges, baseline data that go back only to the early twentieth century. “That’s all [the data] we have to extrapolate to a situation of one in ten thousand years,” says flood management engineer Jos Dijkman, referring to the need to design infrastructure to cope with millennial-scale events such as the most extreme flooding. “Such an extrapolation is by definition uncertain, and you can go into all sorts of statistical methods and techniques to fine-tune that prediction.” Dijkman works for Delft Hydraulics, a Dutch company that has positioned itself as a leader in water management strategies.

## Ground Control

Dijkman says the country’s engineering community has been moving away from a dependence on solid, immutable defenses. Designers have increasingly been looking to the natural landscape to mitigate the impact of flooding on developed areas, freeing up regions such as marshlands to take on excess water temporarily and so lessen a tendency to continue raising the height of levees as an exclusive means of enhancing protection. An example of this policy goes by the name “Room for the Rhine,” which combines engineering principles with research into the factors affecting the health of floodplains, such as the relationship between vegetation and water quality. In places where the setting back of a dike has not been possible, the Dutch also reserve “green” rivers, areas between dikes where water flows only during floods.

“For the old-fashioned way of building a gigantic floodway, you don’t necessarily have to know the [wetlands] system in all the details” says Dijkman. “If you want to develop a wetland that will absorb the energy of flood surge, you’d better know in detail what the processes are that drive the formation of these wetlands.”

Following the devastating flood of 1953, Dutch engineers also began to develop a new generation of tough, synthetic textiles that could be used to anchor earthen levees from below, preventing movement of the soil and even the penetration of water. A domestic manufacturer, Nicolon BV, emerged as one of the leaders in this field, eventually setting up an American operation in Georgia to serve the U.S. market. In 1991, Nicolon joined forces with North Carolina–based Mirafi, which had been experimenting with even more sophisticated geosynthetic fabrics since the late 1960s.

This technology was used to refurbish and upgrade parts of the New Orleans levee system as recently as the summer of 2005. On that occasion, the U.S. Army Corps of Engineers used a 900-foot section to compare the effectiveness of three Mirafi products—an impermeable geosynthetic textile and two types of a more loosely woven material known as geogrid. Strain-monitoring gauges were installed as part of this work. Although the geogrids lent slightly greater stability to the soil, the geotextiles perfomed nearly as well and saved nearly $340,000 (46%) over the cost of the geogrid.

## Feedback from Fiber Optics

Sheer physical mass will never be sufficient to protect against waters that would flood. Aftab Mufti, president of the Intelligent Sensing for Innovative Structures (ISIS) Canada Research Network, compares the situation of today’s levee builders with one faced by a previous generation of aircraft designers. Prior to World War II, planes were built and flown without much attention to the specifics of performance, so that revisions to details such as wing span or tail height were being carried out constantly, based on in-service flight reports. But the push for high-performance military aircraft accelerated the emergence of a design philosophy that was premised primarily on theory and modeling, rather than simply building something and seeing if it would fly.

Today’s aerospace engineers would be loathe to put something in the air that had not been modeled extensively on computers and in wind tunnels, using flight data obtained using avionics, so that the final working product differs little from the prototype. Mufti regards civil engineers as being ready to make the same leap in their field, after many generations of building structures that are far less modeled and monitored than they could be. He says the civil engineering discipline will have to develop “civionics” as the aerospace engineering has developed avionics to be able to monitor the health of civil engineering structures.

More specifically, Mufti endorses the use of electronic and fiber-optic sensors to assess changes in the geometry and forces within a built structure, such as a bridge, a dam, or a levee. These sensors can take advantage of time domain reflectometry (TDR), in which light signals sent through a fiber-optic cable (set, for example, into the soil of an embankment) with any interruption reflect movement that can be readily located. Over time, Mufti says, these readings can provide invaluable insight into how well a structure is holding up.

“What you get out of this is data which you can use to improve your designs in the future,” he says, adding that these data can likewise be applied to future construction regulations. “Our codes at the moment are approximate, therefore conservative. We work in the laboratory and do the testing and monitoring of the structures and materials in the laboratory. Now what we’re finding is that structures and materials behave and age in real life quite differently than what we are seeing in the laboratory.”

Among the leading firms collecting such TDR data is Kane GeoTech, based in Stockton, California, which has carried out much of its work on the levee systems in the floodplain around Sacramento. The most likely model for use in New Orleans is a system deployed since 2002 by Kane GeoTech to measure pore pressures and seepage beneath a levee in the Sacramento River Delta. Vibrating wire piezometers measure water levels in the adjoining river, as well as pressures underneath the levee structure, correcting the latter against parallel measurements of barometric pressure above. These data are collected every hour, and can be downloaded by an inspector to a handheld computer from onsite monitoring stations.

Kane GeoTech has also installed a slightly more sophisticated system for railroad tracks that run along coastal cliffs for trains operated by the North County Transit District in San Diego. Here pulses are sent along cables every four minutes, and any spikes in the signal that would indicate ground movement are sent to a central office, which can immediately dispatch personnel to check out the situation.

Kane GeoTech representatives have suggested that similar TDR sensor cables could be installed in damaged New Orleans levees as they are being rebuilt, thereby minimizing the cost of introducing a similar monitoring system to this area. Given the communications technology that is now available, this instrumentation could well include modems that would transmit the resulting data over the Internet.

## Innovation of Another Sort

One thing that’s certain is that Hurricane Katrina exposed the limitations of the traditional approach to levee building, as was obvious to a national panel of experts investigating firsthand how the storm surge after the hurricane caused the New Orleans structures to fail. The panel noted several instances where simple improvements could be made. For instance, a great deal of damage occurred when water overtopping the levees created waterfalls that tumbled over the normally dry sides of these structures. These steady cascades created “scour holes” that weakened levee foundations. This problem could be mitigated by placing concrete protective aprons at points where such waterfalls could occur.

Panelist Tom Zimmie, acting chairman of the civil and environmental engineering department of Rensselaer Polytechnic Institute, acknowledges that solutions to these problems may prove to be more expensive than even the most ambitious rebuilding effort will accommodate. But he argues that the scale of the project would make even the most modest improvements well worthwhile. “You’re talking about millions and millions of cubic yards of dirt,” he says. “There’s three hundred fifty miles of levees; a lot of them have to be patched up. A small innovation, a small saving, is a big deal.”

Dijkman notes, however, that building and monitoring infrastructure is not sufficient to fully protect against flooding. “A legal framework that requires regular reporting to the government about both the quality of the infrastructure and possible changes in storm conditions ensures that politicians are informed about any deficiencies,” he says. “They can then use that information to appropriate funds to help the flood defenses meet their original objectives.”

Dutch law not only specifies protection levels for flood-prone areas, but also requires levee managers to inspect their levees every five years, taking into account updated storm conditions. Dijkman suggests, “It could be worth considering such legislation in the United States. This could avoid any gap between the information available in the engineering and science community and the political arena.”

## Figures and Tables

**Figure:**
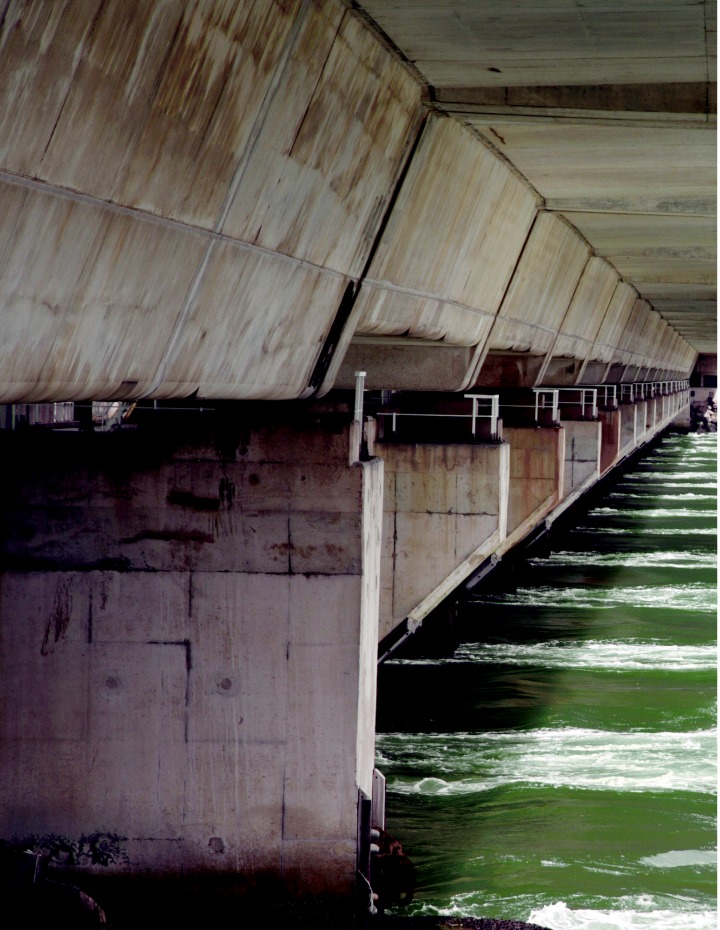


**Figure:**
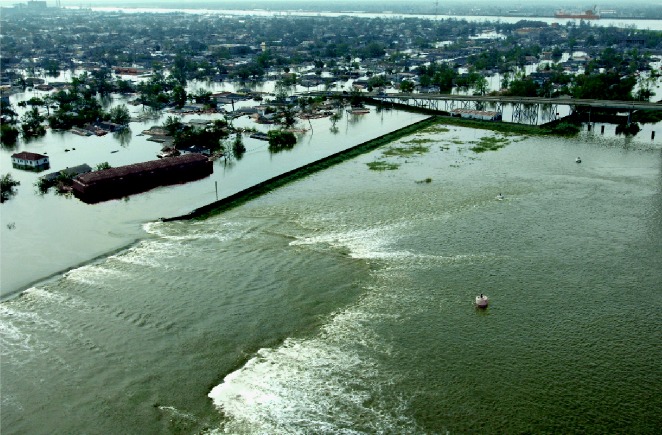
Hope for renewal. Use of innovative construction and maintenance technologies may allow engineers to rebuild the New Orleans levee system (shown here flooding the Ninth Ward on 30 August 2005) stronger than before.
